# Alienation: A useful concept for health inequality research

**DOI:** 10.1177/14034948221085394

**Published:** 2022-05-13

**Authors:** Emil Øversveen, Conor A. Kelly

**Affiliations:** 1 Department of Sociology and Political Science, NTNU, Trondheim, Norway; 2 NTNU, Trondheim, Norway

**Keywords:** Health inequality, psycho-social health, alienation, theory, Marx

## Abstract

**Aims:**

While Marxist class analysis has strongly influenced the development of health inequality research, other aspects of Marx’s theory have received less attention. Among the most relevant of Marx’s theoretical contributions for social inequalities in health is the theory of alienation. As empirical applications of the theory of alienation are currently scarce, the purpose of this commentary is to invigorate interest in alienation theory within the field of health inequality research by demonstrating its potential to illuminate the relationship between social inequality, psycho-social affects and health outcomes.

**Results:**

Alienation theory describes how the class structure of capitalist societies creates experiences of powerlessness, estrangement and isolation. These experiences are further posited as emerging from the exploitation of labour, thus connecting social inequality to psychological wellbeing. Alienation theory is particularly compatible with psycho-social explanations of health inequalities, which similarly posits that social inequality affects health through psychological mechanisms. We argue that alienation theory contributes in three ways to health inequality research: a) by suggesting potential mechanisms and offering predictions that may be put to use in empirical research, b) by providing a potential explanation of the welfare state paradox, and c) by situating the psycho-social determinants of health within a critical analysis of the sources of social inequality in capitalist society.

**Conclusions:**

**Alienation theory may provide a more textured understanding of the relationship between inequality and psycho-social health, while also foregrounding issues of class, power and exploitation that are often absent from psycho-social explanations**.

## Introduction

Marxism and health inequality research has a long and complicated relationship. The history of this relationship dates to 1845, with the publication of Friedrich Engels’ *The Conditions of the Working Class in England* [1]. Containing detailed documentation of social inequities in life expectancy and morbidity following the Industrial Revolution, Engels’ work is not only considered a pioneering work within the field of health inequality research, but also in the development of Marxism more generally [2]. Furthermore, Marxist class theory was cited by the authors of the Black report, who advanced a ‘materialist’ explanation of health inequalities as the result of structurally determined inequities in production and consumption between social classes [3].

In the decades since the Black report was published, the field of health inequality research has generally moved away from explicit references to social class and exploitations towards more theoretically and politically neutral operationalizations of socioeconomic status (SES) [4, 5]. Today, Marx’s influence is particularly visible within what may be loosely termed ‘critical’ health inequality perspectives: programmes that seek to advance more theoretically and politically radical understandings than what is afforded by ‘mainstream’ approaches. Examples of critical health inequality perspectives include neo-Marxist class analysis [4], Scambler’s greedy bastard hypothesis and critical realist asset flow theory [5], and Waitzkin’s work on imperialism and global health [2]. These perspectives generally argue for replacing measures of socioeconomic position with more structurally oriented operationalizations of social class, paying more attention to issues of power, oppression and exploitation, and moving away from policy-oriented activism towards a more radical political agenda.

While Marxist class theory continues to exert a powerful influence on health inequality research, other key concepts in Marx’s critical theory have received comparatively limited attention. Among the most influential of these concepts is *alienation*, which describes how the capitalist mode of production engenders feelings of estrangement, powerlessness and isolation at both the individual and societal level. As Crinson and Yuill have argued [6, 7], alienation theory may benefit health inequality research by bridging the gap between psycho-social and materialist frameworks. While material explanations highlight the unequal distribution of wealth and its material consequences, psycho-social explanations argue that the association between social position and health can be explained by the negative emotions engendered by living in unequal societies, thereby adding an important psychological dimension to the understanding of health inequalities [8-11]. However, psycho-social explanations have also been criticized for furthering a methodological individualism that reduces structural injustice to a matter of individual psychology, and for failing to connect subjective states to the economic factors highlighted in material frameworks [4].

This commentary seeks to reinvigorate interest in alienation theory by demonstrating its empirical, theoretical and practical relevance for health inequality research. Alienation theory, we claim, enhances our understanding of the subjective experiences of deprivation by resituating them within a critical understanding of the political and economic arrangements that generate inequities on a structural level. We begin by providing a brief review of psycho-social explanations, before examining how alienation theory may enhance present understandings of the relationship between social inequality, psychological states and health. We argue that alienation theory may contribute in three ways: by a) suggesting potential mechanisms mediating the relationship between social class and health, b) illuminating the ‘paradoxical’ persistence of health inequalities in developed welfare states, and by c) situating the psycho-social determinants of health within a general theory of class and inequality in capitalist societies.

## Psycho-social explanations

Psycho-social explanations postulate that social inequality induces anxiety, depression, stress, and other emotional states that generate unfavourable health outcomes, thus creating a causal relationship between social position and health. While research on the psycho-social causes of health inequalities is arguably too diverse as to be described as a unified theory, Elstad [12] has identified three core assumptions: 1) that the unequal distribution of stress is an important determinant of health inequalities in affluent societies, 2) that stress is significantly influenced by the individual’s social relations, and 3) that the quality of the latter is influenced by overall levels of social inequity. Emotional distress may affect biological processes in the body directly (e.g. the association between stress and heart disease), or indirectly (e.g. the association between stress and risk behaviours) [13-17]. Typical psycho-social determinants include experiences of relative deprivation, work-related stress, lack of job autonomy, lack of social capital, and experiences of discrimination and stigma, as well as the negative life events and stressors that having a low SES may entail. A great part of the appeal of psycho-social explanations lies in their potential for explaining the growth of health inequalities in post-war Western societies, due to the suggestion that social inequality will continue to damage health even in the absence of directly health-damaging material conditions [8, 9].

While psycho-social explanations remain prevalent within health inequality research, they have also faced criticism. Neo-materialist researchers argue that psycho-social explanations, through their emphasis on subjective status perceptions, underestimate the importance of objective material inequalities and oppressive social institutions [4]. From a practical perspective, the argument goes, psycho-social explanations are prone to appropriation by regressive and victim-blaming political agendas that substitute real redistribution for cultural interventions [6]. In his review of theoretical explanations of the ‘welfare paradox’, Mackenbach further argues that psycho-social explanations fail to explain the fact that health inequalities tend to be larger in Scandinavian welfare states than in countries with less egalitarian income distributions [10]. Similarly to many other health inequality theories, psycho-social explanations also bracket the question of how social inequality arises in the first place, arguably blunting the perspective’s theoretical and critical edge [11]. Finally, empirical applications of psycho-social frameworks have been criticized for lacking methodological and theoretical sophistication, and for failing to properly assess questions of power, class and exploitation [18].

Based on this exposition, we posit that psycho-social theory – while providing a plausible account of the relationship between income inequality, mental states and health outcomes at the individual level – faces numerous theoretical challenges, several of which are related to the failure to establish a connection between subjective affects and objective social structures. These issues can be fruitfully addressed by integrating insights from alienation theory, which explains feelings of isolation, despondency, powerlessness and resentment as the result of the class structure of capitalist societies. To prepare this argument, the next section lays out alienation theory’s main tenets.

## Alienation theory

Marxist alienation theory^1^ begins with the premise that humans are productive beings capable of transforming the world around us to fulfil our various needs and desires [19, 20]. While all animals interact with the world around them as a fundamental prerequisite for survival, humans are distinguished by our capacity to create outcomes that we have consciously conceived prior to these interactions, that is, to *produce.* Production is therefore not only a means of survival, but also the chief mechanism by which we relate to nature, to other people and to ourselves. As the primary expression of human agency, Marxism therefore accords productive activity – labour – a central position in the development of human subjectivity, society and history [21].

Under capitalism, people’s capacity for labour is transformed into a commodity – labour power – which is then sold for a wage. From the commodification of labour arises the primary class division of capitalist society: between those that make their living by *selling* their labour power (the working class) and those that purchase labour power and put it to work in production (the capitalist class). Without disregarding the complexity of the socioeconomic structure in contemporary capitalist societies, Marxist class theory posits ownership and control over productive assets – for example, raw materials, machinery, land or financial resources – as the main determinant of class position. Put simply, workers are compelled to sell their labour power because they lack the assets needed to produce independently, whereas ownership of the means of production allows capitalists to control the production process to their own benefit [22]. Purchasing labour power also enables capitalists to appropriate the surplus value created by the workers, a process referred to as *exploitation*. Distinguishing Marxist class analysis from other theories of class, the exploitation concept advances that the welfare of the privileged depends on the deprivation and exclusion of others, producing a class structure characterized by domination and conflict [21, 23, 24].

Alienation arises from the exploitation of labour, and can be defined as a process in which the results of productive activity are *appropriated* and *transformed* into capital. In Marx’s usage of the term, *capital* refers not only to the material results of production (i.e. wealth), but also to a logic of social development that both conditions and is reproduced through the production process. According to Marx, capitalism differs from previous economic systems in that production is not generally undertaken with the aim of fulfilling human needs directly, but rather producing abstract economic value [21, 25]. This gives capitalism an explosive drive towards social and technological development, with revolutionary consequences for society as a whole: namely, the unprecedented and unrivalled increase of humanity’s social power; its technological and scientific mastery over nature; and a continual upheaval of traditional cultural forms. Due to the exploitation of labour, however, ‘the monstrous objective power’ created by social production ‘belongs not to the worker, but to the personified conditions of production, i.e. to capital’ [25, p. 832]. As the commodification and alienation of labour becomes generalized, the results of production – including society itself – are created in the form of an external reality that stands opposite the individual as an alien, unknowable and uncontrollable force. For the individual worker, alienation engenders feelings of isolation, fatalism and powerlessness, and a sense of disconnect from the society to which they belong and contribute. The effects of alienation are not contained within the sphere of production, however, but ultimately extend to society as a whole. According to Marx, capitalism is distinguished from previous social formations in that economic activity is mediated through abstract economic categories rather than direct cooperation; capitalist development thus tends to substitute direct relations with an abstract and quasi-natural social order [21]. In other words, while capitalist domination is ultimately rooted in the exploitation of labour, human agency appears to be not only restricted by direct coercion by other people, but also by a set of impersonal and seemingly universal imperatives [26]. Due to the alienation of labour, therefore, what is objectively an increase in humanity’s social power and interdependence is subjectively experienced as its opposite, namely as powerlessness and isolation [20].

## Alienation and health inequalities: Three contributions

In this section, we will specify three contributions alienation theory can make to health inequality scholarship. Specifically, we 1) illustrate how alienation theory may be used to supplement psycho-social theory by suggesting additional meditating mechanisms between social class and mental states, 2) apply these insights to the Nordic setting to reassess the welfare and Nordic ‘paradoxes’ and 3) discuss how alienation theory deepens and fine-grains the understanding of the psycho-social determinants of health inequalities by situating these mechanisms within a general theory of the class structure of capitalist societies

Alienation theory might firstly contribute by suggesting a number of mechanisms that can be readily operationalized and investigated in empirical research. In the *Economic and Philosophic Manuscripts*, Marx notes how the alienation of labour – produced and intensified by the unequal distribution of economic resources – estranges the worker from the product of their labour, the act of production, from other people, and from themselves [27]. Several work-related mechanisms are suggested in this brief description. These include job satisfaction, autonomy, experiences of work-related meaningfulness, work ability, job insecurity and influence over work-related decision making, several of which have been linked to health outcomes in previous studies [28-33]. The salience of alienation for psycho-social health is also supported by studies that have found associations between alienation and social class, experiences of exploitation, poor work relations and lack of workplace democracy, which in turn have negative consequences for mental health and overall wellbeing [32, 34, 35]. A 2021 European study found that alienation, while most prevalent in declining blue-collar occupations, is still experienced by a sizeable number of employees, further demonstrating alienation’s relevance in contemporary European societies [32].

Alienation is not only confined to the workplace, however, but may also be fruitfully employed to explain general levels of psychological wellbeing as determined by the unequal distribution of social power, commodification processes, the displacement and deskilling of labour due to technological automation and the privatization of previously public institutions and services. While it is outside the scope of a theoretical commentary to consider the empirical magnitude of alienation, we may predict that alienation is likely to be increased if rapid social development coincides with increased social inequities, particularly if these inequities are driven by the concentration of wealth and power in society’s upper strata. Within societies, the effects of alienation are likely to be most acute for people working in highly technologized and standardized industries, as well as the precarious populations most directly exposed to market forces.

An important part of psycho-social frameworks’ appeal lies in their ability to illuminate the ‘paradoxical’ persistence of health inequalities in Western social democracies. While we maintain that material factors also remain salient in developed welfare states, alienation theory has the potential to illuminate why health inequalities may persist or even increase in the face of generally improved living conditions. Traditional formulations of the ‘welfare’ and ‘Nordic’ paradoxes often presuppose that social inequality is primarily a matter of the distribution of material resources, typically income and wealth [10]. However, social inequality also concerns the question of who has a say in determining the shape and direction of society, a question in which control over production is of central importance. By foregrounding the latter, alienation theory expands the sometimes one-sided focus on economic distribution to include issues of power, recognition, identity, democracy and social relationships. These inequalities may operate relatively independently of the resource inequalities typically considered in health inequality research, providing a possible explanation for why the relationship between economic inequality and health inequality is not linear.

The contribution of alienation theory is not limited to expanding the space of potential mediators in the relationship between social class and mental states as a simple appendage to psycho-social theory, but also relates the affects highlighted in psycho-social frameworks to the social organization of production, thus providing a more textured and explanatory account than is traditionally afforded by psycho-social explanations. Despite the fact that psycho-social frameworks often posit economic inequality as the causal root of empirical health inequalities, the question of how and why economic inequality is manifested and perpetuated is generally bracketed [10]. This risks naturalizing social inequality by presenting it as both an a priori and spontaneously arising phenomenon, obscuring the specific processes through which inequalities are produced and sustained. Alienation theory, and Marxism more generally, addresses this shortcoming by situating the psycho-social determinants of health inequalities within a general theory of capitalist societies. Psycho-social mechanisms – for example the experience of disenfranchisement, anxiety precipitated by employment precarity, social isolation, feelings of hopelessness or powerlessness, and the lack of resources to cope with these states – are therefore not explained primarily with reference to subjective perceptions, as psycho-social explanations have occasionally been criticized for doing [6], but rather to the ‘upstream’ economic and political developments highlighted in structurally oriented health inequality research. Thus, alienation theory expands the ‘spirit level’ hypothesis that inequality generates ‘unhealthy’ societies by pinpointing the concrete mechanisms through which this relationship manifests, providing a more specific and arguably more sophisticated explanation than general references to often vaguely defined concepts of social solidarity and integration [18]. Applying alienation theory to psycho-social health inequality research may therefore address one of the main critiques of health inequality research, namely its reluctance to position empirical findings of health inequalities within a more general critique of class, power and exploitation [4, 5].

Although we have largely focused on the implications of alienation theory for the working class, the theory has comparable albeit dampened implications for other classes and social groups in capitalist societies as well. We propose that the health-related effects explained by alienation theory can likewise be observed even among groups like small business owners, managers, and so on – the sources and impact of psycho-social factors will, in concert with materialist explanations, increase as a function of decreasing class. We therefore argue that our general treatment should not be readas a limitation of alienation theory’s demographic remit, but rather a reflection of our decision to favour a more abstract-level discussion.

## Conclusion

In this commentary, we have argued that alienation theory has a potential to elucidate the interactions between the structural sources of social inequality and psycho-social determinants of health outcomes. Alienation theory centres the tendency of capitalist societies to generate experiences of powerlessness, loss of meaning, isolation and despair, suggesting a causal link between material conditions and psycho-social states that may be explored in empirical research. As part of our argument, we have presented three ways in which alienation theory can improve and deepen our understanding of health inequalities: 1) by revealing potential mechanisms and predictions that may be employed in empirical research, 2) by suggesting a potential explanation of the welfare state paradox, and 3) by situating psycho-social determinants of health within the structural mechanisms that generate inequity in capitalist societies. Thus, we hope to have demonstrated the potential of alienation theory to foreground issues of class, power and exploitation that are often absent from psycho-social explanations, without compromising the latter’s capacity to capture nuanced affective experiences relevant for understanding health outcomes.

## Notes

1 This section provides a highly condensed version of a Marxist theory of alienation developed by the first author. For an extended elaboration and justification, see Øversveen, 2021.
